# College and Hospital Notes

**Published:** 1910-06

**Authors:** 


### COLLEGE AND HOSPITAL NOTES.

THE University of Buffalo is celebrating commencement week as these pages are going through the press. The Alumni Association began its work on Tuesday, May 24, with clinics at 10 o'clock. The lectures in the Harrington series were delivered by Dr. J. Clarence Webster, of Chicago,—the first on Tuesday, the second on Wednesday, and the third on Thursday. Clinics were continued during Wednesday and Thursday, and Thursday evening a complimentary dinner was tendered to Dr. Matthew D. Mann, who retires from active work as a teacher with the close of this academic year. The sixty-fifth commencement exercises of all the departments were held at the Teck Theater at 11 o'clock Thursday morning—the address being delivered by Dr. Mann. We expect to publish a detailed account of the proceedings in the July edition of this magazine.

A Demonstration of practical nursing by the graduating class, of the Buffalo General Hospital, in the operating room was given Wednesday, May 11, 1910, at four o'clock. Dr. M. D. Mann presided.

The following program was carried out, and proved efficiency on the part of the nurses.

1. Giving a mustard foot-bath in bed, to reduce temperature.

2. Changing the mattress and bed clothes of a helpless patient.

3. A patient suffering from heart disease, made comfortable in sitting position.

4. Bathing a baby; Sloane maternity method.

5. First aid to injured; improvising a temporary support for a broken leg; or improvising a stretcher to remove the patient.

6. Croup kettle treatment for bronchitis, (a) In the hospital; (b) In a tenement house.

